# India Research Management Initiative (IRMI) – an initiative for building research capacity in India

**DOI:** 10.12688/wellcomeopenres.15073.2

**Published:** 2019-04-10

**Authors:** Savita Ayyar, Shahid Jameel

**Affiliations:** 1 Jaquaranda Tree, Bengaluru, Karnataka, 560064, India; 2 Wellcome Trust/DBT India Alliance, New Delhi, 110025, India

**Keywords:** Research, Research Management, India, Extramural Funding, RMA, Scientific Administration, Science careers, Professionalization, IRMI

## Abstract

Research and innovation are growing in India with significant investments being made towards institutions, researchers and research infrastructure. Although still under 1% of GDP, funding for science and technology in India has increased each year for over two decades. There is also increasing realization that public funding for research should be supplemented with that from industry and philanthropy.

Like their counterparts worldwide, Indian researchers require access to professional research management support at their institutions to fully leverage emerging scientific opportunities and collaborations. However, there are currently significant  gaps in the research management support available to these researchers and this has implications for research in India.

The India Research Management Initiative (IRMI) was launched by the Wellcome Trust/DBT (Department of Biotechnology, Government of India) India Alliance (hereafter India Alliance) in February 2018 to narrow these gaps. A 12-month pilot phase has enabled conversations across multiple stakeholders. In this Open Letter, we share some insights from the IRMI pilot phase, which could aid systemic development and scaling up of research management as a professional support service across India. We anticipate these will stimulate dialogue and guide future policy and interventions towards building robust research and innovation ecosystems in India.

## Disclaimer

The views expressed in this article are those of the authors. Publication in Wellcome Open Research does not imply endorsement by Wellcome.

## Background

Research and Innovation in India is supported through significant investments from the Government of India, international agencies and more recently from the private sector. The National Science and Technology Management Information System (NSTMIS) Division lists nearly 7000 research institutions in India, including Central and State Universities, Central Government research institutions, Public sector and Private sector institutions and others ^1^. Over 50% of research in India is supported with public funds from the Government of India, channelled through sources including the Department of Biotechnology (DBT), Department of Science and Technology (DST), Council for Scientific and Industrial Research (CSIR), Indian Council of Medical Research (ICMR) and Department of Atomic Energy (DAE) ^1– 3^.

Research support from the Government of India to Indian investigators includes competitive extramural funding from government agencies, via a wide range of competitive grants, fellowships and international collaborative funding schemes ^2^. There are additionally opportunities for research via international funding partnerships such as the Wellcome Trust/DBT India Alliance (hereafter India Alliance), European Molecular Biology Organization (EMBO) and the Human Frontier Science Program (HFSP). Several philanthropic organizations including the Bill and Melinda Gates Foundation, Howard Hughes Medical Institute, Simons Foundation, Tata Trusts and Wellcome Trust support investigators and research projects in India.

While robust systems for managing intramural funding to research institutions are in place, corresponding processes for helping Indian researchers compete successfully for extramural funds have lagged behind. The current funding landscape presents both a need and an opportunity for India to develop a sound support base for this purpose.

## About the IRMI Pilot

Research management (RM) systems worldwide have evolved in unique ways, driven by the complexities of research and innovation, the funding landscape and collaborative opportunities ^4– 8^. As an early step towards understanding RM practices in India, the Wellcome Trust, UK commissioned a scoping study in 2016 on research management (RM) in India, which included five Indian research institutions receiving funding from the India Alliance ^9^. The India Alliance subsequently coordinated a panel discussion titled “Research Development Offices: The Need of the Hour” at its 2017 Annual Fellows meeting. Additionally, a voluntary and anonymous survey of India Alliance Fellows was carried out in 2017 to assess existing support for laboratory, data and research management, and research misconduct. Only 18% of respondents in the survey confirmed the presence of a Research Development Office at their institutions ^10^. These early steps highlighted the need for developing and sustaining RM support at Indian research institutions. 

**Figure 1.**
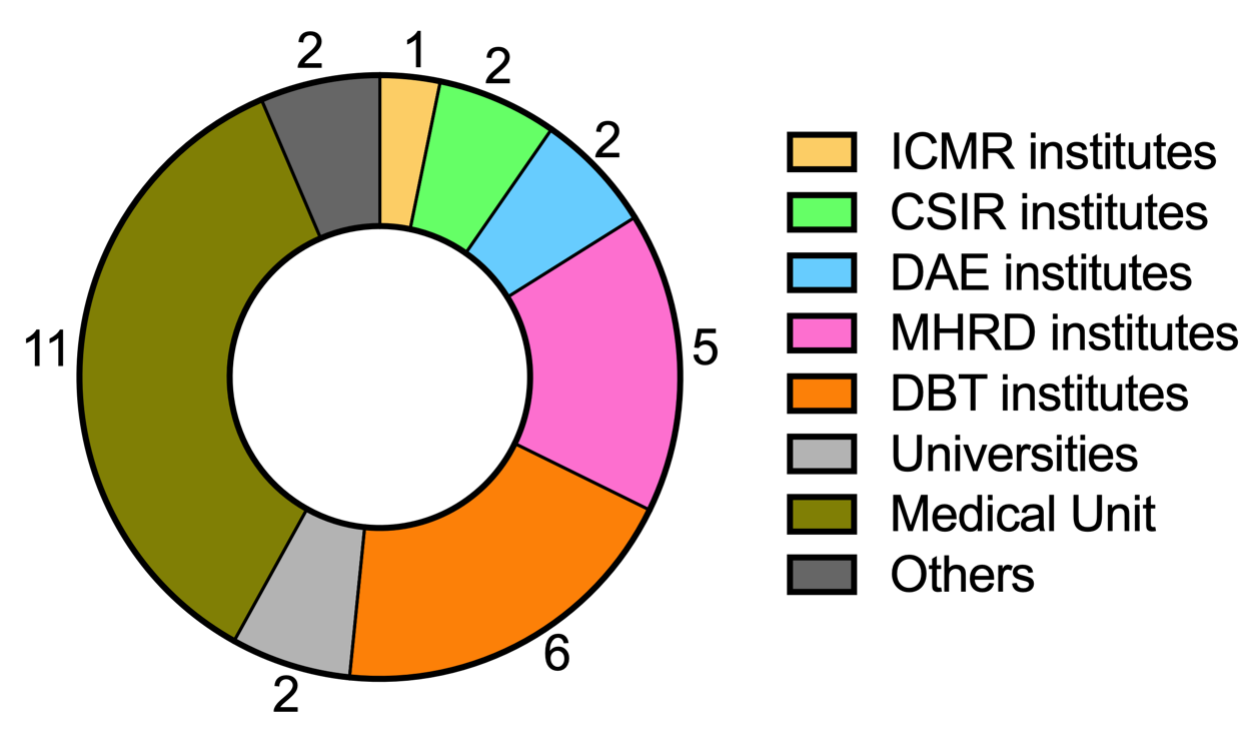
Diversity of institutions engaging with the India Research Management Initiative (IRMI) initiative, including autonomous research institutions of Government of India Departments such as Indian Council of Medical Research (ICMR), Department of Biotechnology (DBT), Department of Atomic Energy (DAE), Ministry of Human Resource Development (MHRD), CSIR; Universities, Medical Centres & associated research units and Others.

**Figure 2.**
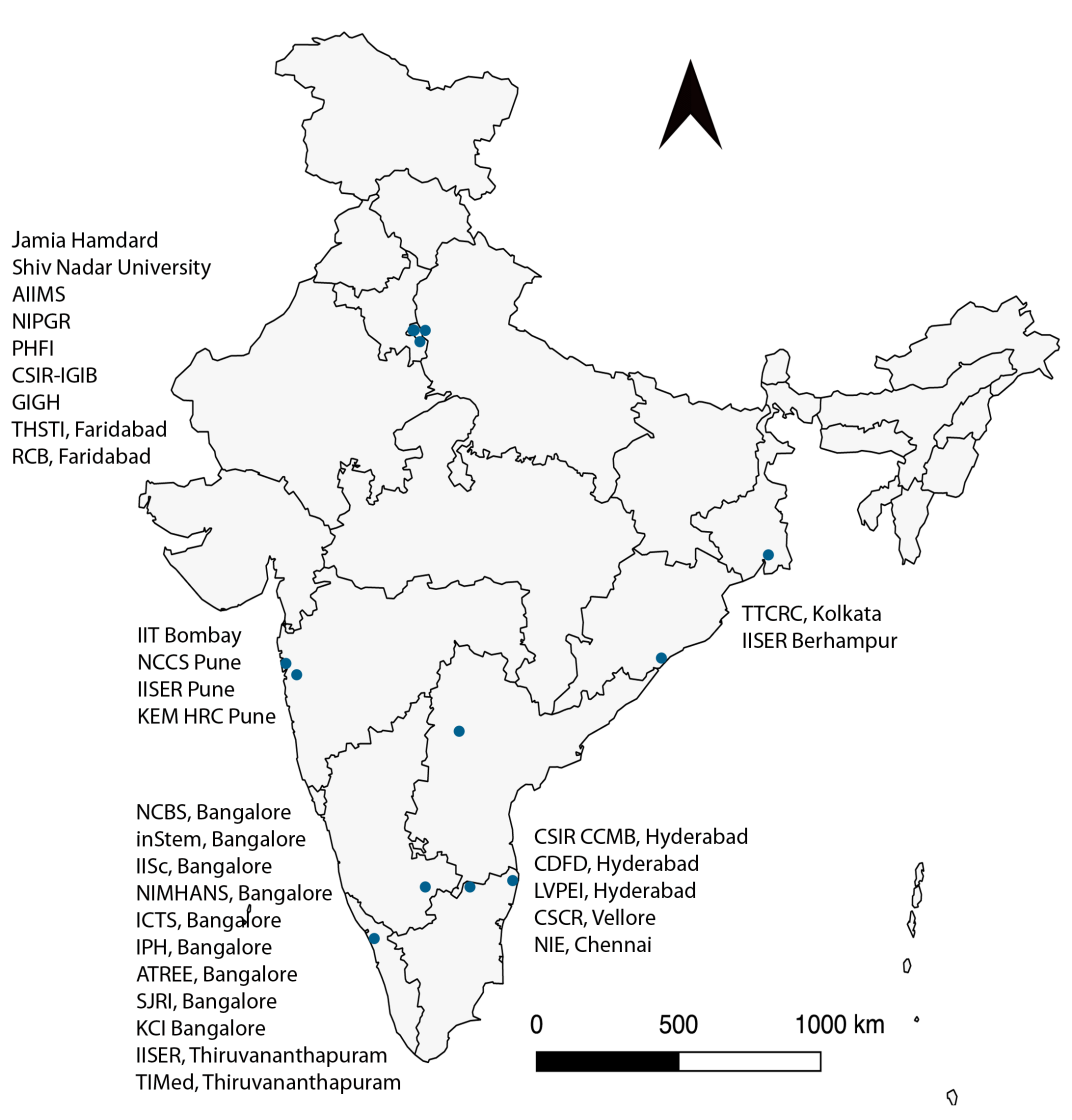
Geographical locations of institutions engaging with the India Research Management Initiative (IRMI) initiative.

Following on from these exercises, the India Alliance formally launched the India Research Management Initiative ( IRMI) in February 2018 as an India-led 12-month pilot study aimed at creating awareness for research management, engaging in dialogue with Indian institutions and building a baseline of information upon which to base future policy and funding opportunities. The IRMI pilot has allowed us access to scientific leadership, faculty members, research managers and administrators at 31 participating institutions ( Figure 1, Figure 2 and Table 1), staff at major research funding agencies in India and members of the international research management community.

**Table 1.** List of Indian institutions engaging with the IRMI initiative.

	Name of research organization
1	Jamia Hamdard, New Delhi
2	National Institute of Epidemiology, Chennai
3	Centre for Stem Cell Research Vellore
4	Regional Centre for Biotechnoloy, Faridabad
5	CSIR- Centre for Cellular and Molecular Biology, Hyderabad
6	Translational Health Sciences and Technology Institute, Faridabad
7	National Centre for Cell Science, Pune
8	Indian Institute of Science Education and Research, Berhampur
9	Indian Institute of Science Education and Research, Pune
10	Indian Institute of Science Education and Research, Thiruvananthapuram
11	KEM Hospital and Research Centre, Pune
12	Institute for Stem Cell Biology and Regenerative Medicine, Bangalore
13	National Centre for Biological Sciences, Bangalore
14	Indian Institute of Science, Bangalore
15	George Institute for Global Health, New Delhi
16	Shiv Nadar University, Uttar Pradesh
17	All India Institute of Medical Sciences, New Delhi
18	Indian Institute of Technology, Bombay
19	Sri Chitra Tirunal Institute for Medical Sciences and Technology, Thiruvananthapuram
20	Public Health Foundation of India, New Delhi
21	National Institute of Plant Genome Research, Delhi
22	CSIR- Institute of Genomics and Integrative Biology, Delhi
23	St Johns Research Institute, Bangalore
24	Ashoka Trust for Research in Ecology and the Environment, Bangalore
25	International Centre for Theoretical Sciences, Bangalore
26	Kidwai Cancer Institute, Bangalore
27	Institute of Public Health, Bangalore
28	National Institute of Mental Health and Neurosciences, Bangalore
29	Tata Translational Cancer Research Centre, Tata Medical Centre Kolkata
30	Centre for DNA Fingerprinting and Diagnostics, Hyderabad
31	LV Prasad Eye Institute, Hyderabad

CSIR - Council of Scientific and Industrial Research

We interacted with individuals in roles supporting grant management, project management, scientific outreach, innovation management, academic programs, financial management, operations, policy development and ethics in India, hereafter defined as **Research Managers and Administrators (RMAs).**

Conversations with institutions were centred on a framework of three themes: (i) Leadership support for research management, (ii) sustainability of research offices, and (iii) career development needs for RMAs. Discussions with stakeholders were conducted via site visits, audio and video calls, IRMI workshops, panel discussions and social media. To gather funding agency inputs on pre-award and post-award matters, staff feedback from the India Alliance was collected for the quality of grants processes followed at institutions. These conversations have allowed us to build an initial picture of expectations, constraints and requirements for various stakeholders.

## Insights from the IRMI pilot

### A broader working definition of RM is required for India

Indian institutions encourage their researchers to raise funds from extramural sources including the Government of India and other funders, both to further research and as peer-reviewed endorsement of their research. Several institutions therefore have in place dedicated grant management offices, such as the Project Management and Evaluations (PME) Cells at research institutions of the Council of Scientific and Industrial Research (CSIR), wherein support services are largely centred around financial management and reporting on extramural grants. Such offices need to widen their scope, incorporate proactive approaches and provide more responsive support to researchers.

India now requires a more comprehensive and inclusive definition of RM, which is also acceptable across institutions as well as funders. A more contemporary view of RM includes grant management at pre-award and post-award stages, partnership building at national and international levels, outreach to funding agencies, ethics, policy, managing team-science, impact analysis and others. Indian institutions developing their RM activities would benefit from taking this broader international scope into account for creating well-structured support services which address specific research needs.

### The beginnings of wider RM in India

In the last decade, a small number of research institutions have taken steps to create science-led RM structures that extend beyond financial management. The National Centre for Biological Sciences (NCBS) in Bengaluru, the Translational Health Science and Technology Institute (THSTI) in Faridabad and Indian Institute of Science Education and Research (IISER) in Pune are pioneers, with operations including international activities, partnership building, grants management at pre- and post-award stages, outreach and ethics. These institutions have a track record of successfully attracting and managing diverse sources of external funding, including the highly competitive India Alliance fellowships. Researchers and the leadership at these institutions regard support from research offices to be crucial for their success, and include these in future planning.

Other government and privately funded institutions have also started investing more broadly in RM. Examples of these are the National Centre for Cell Science (NCCS) in Pune, Centre for Stem Cell Research (CSCR) in Vellore, Public Health Foundation of India (PHFI) in New Delhi, Shiv Nadar University in Delhi-National Capital Region (NCR), George Institute of Global Health (GIGH) in New Delhi, Tata Translational Cancer Research Centre (TTCRC) in Kolkata and Ashoka Trust for Research in Ecology and the Environment (ATREE) in Bengaluru.

At some of these institutions, development of *de novo* RM structures has been driven by the lateral movement of scientific administrators trained at funding agencies including the Wellcome Trust, Department of Biotechnology and India Alliance. These professionals have transmitted funding best practices to their new organizations and have worked in close collaboration with visionary and supportive management teams to build research offices from first principles. These are promising developments, which should be amplified across many more institutions.

### Building new research offices

At present, Indian investigators spend a significant fraction of their time on administration, including the time spent on individually following up on their grant submissions and active grants with funding agencies. In the words of an India Alliance staff member, “In the absence of a central office, grant holders are often fighting a lone battle. They have to individually follow up with various Departments and scientific leadership at their host institutions as well as funding agencies to ensure that all grant-related requirements are met. While they would prefer to focus on their research programs and mentoring early career staff, much of their time is spent chasing after such tasks”.

Professional research management advice and support can significantly reduce the administrative burden on researchers and improve the effectiveness of funding proposals ^5, 11, 12^. Outreach to funding agencies via a well-functioning centralized office is required for efficiency and creating institutional memory, and would be immensely beneficial to individual researchers, particularly in the context of proactive fundraising from diverse sources.

Institutions should take the initiative to build RM structures to support their unique research priorities. This additionally requires consistently demonstrating the value of RM to researchers and administration alike, to ensure acceptance and long-term sustainability. Leaders should create a climate of trust and actively promote the use of their research offices. This would need to be done in parallel to building capacity in areas such as laboratory management.

Individual researchers at institutions can take an interest in developing their institutional grants offices, and provide inputs and constructive feedback into how such offices could best support their needs. They could also connect with peers across India, via leadership networks, shared administrative structures and platforms such as IndiaBioscience, to explore solutions to issues encountered in creating research offices in India.

### Diversity of Indian research organizations: implications for RM

Research in India spans agricultural, biological, biomedical, chemical, physical, mathematical, earth, engineering and materials sciences, and other disciplines including social sciences. Institutions such as the All India Institute of Medical Sciences (AIIMS) and Indian Institutes of Technology (IITs) impart quality education in medical and engineering disciplines, respectively, and are also well regarded for their research efforts ^1^.

Systemic efforts at boosting RM in India should also take into account the operational sizes and administrative complexities of India’s myriad research institutes and universities ^13, 14^. This currently varies widely, with an average life sciences research institute supporting 30–70 faculty members and the universities, AIIMS, IITs and others having much larger faculty bodies. With changes to funding structures for central and state universities, these higher education centres will also need to establish RM systems suited to their unique requirements ^15^.

### Pre-award grant management- a missing element

Support from a central office at the pre-award stages was found to be available at only 9 of 31 institutions. In many cases, grant applicants did not have access to alerts about forthcoming deadlines, neutral professional advice on funding agency schemes and policies and alignment with institutional focus at the pre-award stage. Lack of awareness also made some researchers sceptical of the value of pre-award support, which was viewed as a hindrance or an administrative bottleneck. 

Institutions have a responsibility to ensure that outgoing grant applications are compliant with legal, financial and ethical requirements. In addition, funders may have their own expectations with respect to matters such as IP, which need to have been considered by the institution. In the absence of structured pre-award services, the leadership at several Indian institutions often do not receive timely support with due diligence on applications, which leads to submission delays and avoidable errors in grant applications (conveyed to us by India Alliance staff).

The lack of proactive pre-award support can compromise both the ability of Indian researchers to identify and seek funding in a timely manner and institutional benefits from pre-award due-diligence and proper budgeting for grant proposals. This would feed forward into the ability of investigators to manage their grants in alignment with agency norms. This aspect of RM will need to be addressed, both from the perspective of changing attitudes and in developing in the required professional support at Indian institutions.

### Team-science: reducing the administrative burden on investigators

Indian researchers are now increasingly participating in complex multi-institutional, often international, team-science projects to address major research questions. With India contributing to international consortia such as EMBO, HFSP and others, Indian researchers have an opportunity to participate and compete at a global level. Managing collaborations requires attention to several administrative considerations, both at pre-award and post-award stages, including budget support and due-diligence at the point of grant submission, project management, regular communication between partners, joint reporting responsibilities, IP management and cross-institutional integration of funding systems and requirements. Such activities would benefit from dedicated RM support for all collaborators, to reduce administrative burden on the investigators and facilitate seamless integration across all participating national and international stakeholders ^16– 19^.

Team-science efforts in India are being funded from both local and international sources and Indian institutions should be willing to request and justify direct resources for RM personnel on grants supporting team-science, rather than expecting their investigators to take care of all administrative requirements.

### Sustainability of careers

India has a substantial pool of early career researchers trained to the PhD and postdoctoral levels. With limited academic positions, scientific administration at funding agencies and research institutions is emerging as an attractive career option. In parallel, there is an expectation from researchers that professionals with “blended” scientific and RM skills will be required to drive a wave of change within current administrative structures at their respective institutions ^20^.

Scaling up RM in India will require the creation of long-term employment opportunities and career structures for RMAs at research institutions across the country. The availability of RM jobs in Indian research institutions should become the norm rather than an exception, as it currently stands. Institutions receiving core-funding from the Government of India face challenges in recruiting RMAs, particularly those with successful academic backgrounds. There is currently no clear path for hiring scientifically trained staff to purely management roles in research organizations supported by the government. Changes to present recruitment norms are required at the policy level to enable government-supported institutions to employ scientifically qualified research managers and create RM structures and roles.

Institutional overheads are globally accepted as a means of supporting research office costs. However, more clarity is needed in India about the use of grant overheads for recruitment of RMAs. It would be beneficial for institutions to work within their respective administrative frameworks to develop clear policies for costing overheads on grant proposals and to utilise a proportion of overheads received towards the recruitment of RMAs.

### Capacity building

With the profession being at an early stage in India, concerted efforts on several fronts are required to prepare and develop an RMA workforce for the next decade. Training programs need to be coordinated in diverse areas of RM, at exploratory, beginner and advanced levels. In order to widen the scope of RM in India, RMAs need access to training modules in several aspects of RM. Training and exchange opportunities should be made available to RMAs in India, potentially through the work of multiple stakeholders.

Individuals with backgrounds in areas such as research, medicine, dentistry and public health would likely play key roles in shaping RM structures for Indian institutions, in a manner that caters to specific institutional requirements and priorities. The profession will hence need to be open to participation from a wider pool of staff with diverse training. Career development programs for Indian RMAs would have to take cognizance of these considerations and incorporate suitable standards.

There are already two RM training programs being offered in India. The Department of Science and Technology (DST) supports training of active scientists at different levels, which does not specifically cater to the career requirements of RMAs. Opening such courses to RMAs would significantly widen the benefits to institutions. Workshops on scientific administration are being supported through the Newton Bhabha Fund and offered by IISER Pune in partnership with the British Council and IndiaBioscience. These workshops, aimed at women candidates wishing to develop careers in scientific administration, have elicited growing interest from the community.

Indian RMAs would also benefit from inclusion in a global community of professionals. IRMI workshops and attendance of Indian delegates at INORMS 2018 were the first opportunities for Indian RMAs to interact with each other and with peers from other parts of the world. There is now a dedicated Linkedin page as an early online community for Indian RMAs. Such networking efforts require nurturing and development. In the longer term, once there is a sizeable RM community in India, it would be beneficial to have a professional association of RMAs, which would be expected to cater to future networking and career development needs of India’s RMAs and for ensuring their connectivity with the international RM community.

### The gender issue

A recent survey has highlighted that in several countries, RM is female dominated ^21^. This is true for India as well. At the IRMI institutions, the majority of RMAs from academic backgrounds are women at early or intermediate stages of their RM careers. The Indian research ecosystem needs to recognise the value of good RM support. It is important for RM to be accepted as a bona-fide profession and not be viewed as an optional route for retaining women with research backgrounds in the workforce, with the risk of their being relegated to ill-defined support roles with unclear paths for career progression.

### Wider participation from other stakeholders

The primary mandate of the India Alliance, which supported the IRMI Pilot, is to enable biomedical research. Conversations during this phase show that RM systems in India need to be inclusive of all areas of science, including social sciences. Beyond IRMI, a wider effort would require collaboration between several funders to support this across disciplines. For maximum impact, the development of RM as a profession in India would require government commitment and participation.

## Conclusions

Indian institutions must now invest in developing a sound RM support base for their investigators. Without such support, the time of a researcher and funds invested in research are not being optimally utilized. The lack of good RM support also risks future growth and the ability to sustainably attract extramural funding from government, private, philanthropic and international sources. Building RM as a viable profession in India will require concurrent creation of sustainable jobs at Indian institutions and training of RM aspirants at different levels. The nascent RMA community in India will benefit from the creation of a formal members association, which can then serve to channelize training, networking and international collaborative opportunities. Such an association could also function as an advocacy group for key funders supporting research in India. With wider participation from RMAs, institutions, mentors and funders, RM can grow considerably in India and make a significant impact on its research and innovation landscape.

## Data availability

No data is associated with this article.
