# Supplementary material for: India Research Management Initiative (IRMI) – an initiative for building research capacity in India
Source: Wellcome Open Res. 2019 Apr 10;4:18. Originally published 2019 Jan 31. [Version 2] doi: 10.12688/wellcomeopenres.15073.2 (PMC6392150; doi:10.12688/wellcomeopenres.15073.2)
Supplement: Peer review history

# Peer review: Reviewer response for version 2

**DOI:** 10.21956/wellcomeopenres.16628.r35282

**Published:** 2019-05-28

**Authors:** Silke Blohm

**Affiliations:** 1 Pan-African University, Gesellschaft für internationale Zusammenarbeit (GIZ), Bonn, Germany

It is good to see that the authors have taken a significant number of peer reviewers’ comments into account, both with regards to establishing a clearer structure as well as a stronger integration of relevant literature. As a result, it reads a lot more fluid.

 The paper is attempting to cover quite a wide range of topics within the subject of research management starting from work into an identified increased need for research management to the development of a profession of RMAs addressing this perceived need. These two parts at times still seem disconnected at times and conclusions are not always evidenced. 

 The need for research management is mainly explained through an increase in external funding and documented through an overall low number of research support structures. This alone does not seem a strong argument for these support structures, even though practice in other countries would indicate the benefit of such offices. 

 The development of the profession of RMAs is explored in more detail. I like the consideration of future career paths within the discipline which will play an important role in the sustainability of such roles and offices. What I would question here is the emphasis on the requirement of an academic background. Looking at regions where research support offices are well established seems to indicate a wide range of backgrounds in successful RMAs which would suggest that this complex field might need a mix of different skills (ranging from various academic (partly non-STEM) backgrounds, legal or financial qualifications, to a wider set of management and partly also leadership skills). In the slightly wider field of higher education management this is often referred to as the ‘third sector’.

 Overall though, the article seems significantly stronger now. As previously said, this seems an important piece of work for a region where research support structures are emerging but are certainly still under researched. Hopefully it will increase the awareness for this subject and lead to an overall strengthening of research at Indian institutions. It also seems to provide a good starting point for future studies and research.

I have read this submission. I believe that I have an appropriate level of expertise to confirm that it is of an acceptable scientific standard.

# Peer review: Reviewer response for version 2

**DOI:** 10.21956/wellcomeopenres.16628.r35285

**Published:** 2019-04-30

**Authors:** Simon Kerridge

**Affiliations:** 1 University of Kent, Canterbury, UK

It is gratifying to see that the authors have taken on board the various reviewers’ comments and have made significant efforts to address them.  The Open Letter is now, I think, much stronger; and makes better use of the evidence base, with far fewer unsubstantiated claims.  The coverage of the literature is also much improved, although perhaps the Colquhoun citation might be placed differently.  I would imagine that rather than being associated with “can significantly reduce the administrative burden on researchers and improve the effectiveness of funding proposals” that it might more naturally be associated with a phrase such as the “viewed as a hindrance or an administrative bottleneck” later in the text.

 One area that I still have an issue with is the asserted perceived need for RMAs to have a scientific background. While this might be the norm for countries developing an RMA infrastructure, it does not appear as prevalent in countries that has a long history of RMA.  But perhaps this is natural evolution over time - an interesting area for research perhaps; similarly with the gender dynamic of the profession.

 Overall the text and structure have been improved and I hope will increase the impact that the Open Letter will have.  In my view this professionalization of RMA is entirely the right direct of travel for building research capacity in India, and I hope that it will be a catalyst for continued change. I would like to congratulate the authors on their efforts.

I have read this submission. I believe that I have an appropriate level of expertise to confirm that it is of an acceptable scientific standard.

# Peer review: Reviewer response for version 2

**DOI:** 10.21956/wellcomeopenres.16628.r35283

**Published:** 2019-04-10

**Authors:** Katrina Lawson

**Affiliations:** 1 Oxford University Clinical Research Unit, Hanoi, Vietnam; 2 University of Oxford, Oxford, UK

This version is much clearer. I think the argument you make for more development of Research Management capacity in India is strong, and the results of the IRMI pilot are also coming through much more clearly in this revised version.   

 I still think that there is a lot more to say about the gender issue (this is an area where more research is needed!), and although I personally disagree that there is a strong need for RMAs to have science backgrounds, the reference to the pilot study data now shows clearly where this expectation has arisen from.  

 I think this Open Letter is really important, and I hope that it leads to more attention for the need to develop Research Management Capacity in India.  

 Congratulations to the author team.

I have read this submission. I believe that I have an appropriate level of expertise to confirm that it is of an acceptable scientific standard.

# Peer review: Reviewer response for version 1

**DOI:** 10.21956/wellcomeopenres.16445.r34744

**Published:** 2019-02-25

**Authors:** L.S. Shashidhara

**Affiliations:** 1 Indian Institute of Science Education and Research, Pune, Pune, Maharashtra, India

In the past ten years, we have seen massive expansion of S&T enterprise in India. Also, in the entire world the way science is pursued is also very different since the beginning of this millennium. All of this demands special category of scientific professionals trained in science management and administration in research/education institutes and universities. They should not only manage day-to-day activities of their organisation, they should also be in the forefront of planning, executing, assessing and communicating (to the policy makers, finance people and public at large) various educational and research activities in S&T. In this context, the survey and its analysis discussed in this article is timely and essential. 

 The article has surveyed a good number of diverse organizations and has outlined the current status of Research management in India. As the article has pointed it out, unfortunately, except for a handful of organizations, in all organizations researchers themselves have to run around to get everything done. Since they are not specialized in these skills, they spend more time, but output is much wanted in terms of quality and quantity. 

 The authors have also made some constructive suggestions on how to improve the situation. The work undertaken by the authors is commendable. 

 However, much of the discussion is on managing grants. Research management goes beyond all this. People with necessary skills of framing policy so that science and its methods are widely used in all policy decisions for improved governance, people with good communication skills, people with administrative skills in setting up laboratories, procuring instruments and reagents, maintenance of equipment, graduate admissions (how to attract and select best students), facilitating national and international collaborations, and science entrepreneurship should be part of a good science management team of any medium size (100+ faculty) to large (400+ faculty) University/research institute. 

 Perhaps, a follow up to this article, authors may consider taking up a survey on how institutional research ecosystem is managed in this country. Perception is, we are not managing well and there is no organizational policy in managing it. A systematic study would give an idea of what India-specific management needs are.

I have read this submission. I believe that I have an appropriate level of expertise to confirm that it is of an acceptable scientific standard.

# Peer review: Reviewer response for version 1

**DOI:** 10.21956/wellcomeopenres.16445.r34741

**Published:** 2019-02-22

**Authors:** Katrina Lawson

**Affiliations:** 1 Oxford University Clinical Research Unit, Hanoi, Vietnam; 2 University of Oxford, Oxford, UK

It is really great to see this report. As with many developing countries, the Research Administration landscape in India is evolving slowly and in a different way that the RA landscape in developed countries. It is rare for resources to be available to investigate and address the infrastructural environment that exists around research support, and the project that has led to the development of IRMI is immensely valuable, as evidenced in this article.

 I think that the article could benefit from some more clear definitions of terms. 

**Intramural vs extramural:** Although it is undoubtedly common in India, I am not clear about what is considered intramural vs extramural funds, and the distinction appears to be significant for this piece. I initially thought that extramural meant competitive funding from outside of India, but now am not sure. How are intramural funds awarded to the researchers? Is it a competitive grant-making process?

**Definition of RM: **The article calls for broader definition of RM, but I’m not sure what the baseline definition is. Is it just financial accounting post-award? The example list of what would be included in a contemporary view of RM is quite all-encompassing – I wonder if there is benefit in providing more clarity around these functions, and perhaps a scale of development. I felt that this section was also a little bit in conflict with the assertion later on that each institution needs to build RM structures that support their unique research priorities.  I think this point is crucial – RM needs to reflect the needs of the research in each context, and there is no single perfect solution.

 In the section about pre-award support, slightly more discussion around the concept of **due diligence** could be helpful. It would be good talk about the needs for institutions to comply with legal and ethical constraints, as well the constraints imposed by funders – which can be significant, particularly in terms of financial control and IP.

 The team science point is very important. You could also specifically mention some of the administrative considerations involved in supporting collaborative research – including IP considerations, shared reporting responsibilities, conflicts of interest, research contract management, and financial reporting and liability for audit.

 I think the point about gender is extremely valuable to make here, and you could make it more strongly. The fact that research administration globally is a female dominated profession is one of the direct reasons that it is undervalued. There is an entire PhD project that could be spent on this particular issue, but for the purposes of this article I would make the point more clearly that Research Administration is undervalued precisely because of gender discrimination. I think it’s not phrased quite right at the moment when you say RM is “a route for retaining scientifically trained women in the workforce”. I think the more obvious point is that RM is being used as a tool to exclude scientifically trained women from the scientific workforce. People should not be training for 15 years to become scientists, and then find that the only research-related work they can get is in the research office doing accounts. I think this also feeds into the capacity building section.  I am not sure of the reason why you seem to arguing that RMAs should preferably be trained scientists. A science background is sometimes an advantage in RM, and other times quite irrelevant. But insisting on a scientific background for the RMAs is probably contributing to the gender imbalance, and the filtering of female scientists into the research office and out of the lab.

 Finally, the case for creating a professional association of RMAs in India is very strongly made here, in particular when considering the need for wider participation from other stakeholders. An RM professional network will be able to broker that support, and advocate for the needs of the research community in relation to RM.

 This review was written under the assumption that the submitted  article is an opinion piece, rather than a scientific report.

I have read this submission. I believe that I have an appropriate level of expertise to confirm that it is of an acceptable scientific standard.

# Peer review: Reviewer response for version 1

**DOI:** 10.21956/wellcomeopenres.16445.r34743

**Published:** 2019-02-20

**Authors:** Silke Blohm

**Affiliations:** 1 Pan-African University, Gesellschaft für internationale Zusammenarbeit (GIZ), Bonn, Germany

The paper describes an important piece of work which seems very timely and is filling a gap within its regional context. The conclusions and recommendations seem compelling and would seem to align with findings/developments in other places. However, this connection is not explicitly made and not evidenced strongly enough. Here drawing on existing studies and publications, e.g. from existing research management associations or some academic studies and papers published, could significantly strengthen the paper.

 The paper gives some important and insightful background on the historical development of research and related funding at Indian universities. This information is useful and would also benefit from references to supporting work and data. The paper then seems to jump too quickly to some initial conclusions before having outlined the foundation for these conclusions. Here a restructure of the order of chapters/paragraphs would help to development a clearer line of argument. It would seem useful to first give an introduction into the Indian HE and research funding landscape before then moving to what seems at the core of the paper, the IRMI pilot.

 The authors have conducted what seems an impressive amount of work on data collection through surveys and individual discussions. The paper gives some insights into findings, overall though could make better use of this data and be more precise about findings and conclusions drawn. 

 While these conclusions made might seem obvious and likely could be supported by data and case studies from similar developments in other regions, those links have not explicitly been drawn and not enough reference has been made to existing work in this area. This would seem a main weakness of this paper which could be addressed by cross-referencing findings and conclusions back to the data collected and to experiences/findings made in other regions/institutions.

 Overall the reader would benefit from a clearer structure which would avoid jumping from observations to conclusions and back. Important aspects to cover would seem:

- a brief historical overview of research and research funding at Indian HEI, 
- an introduction of the pilot this article is based on, the methodology used and why it has been used,
- a brief summary of other work in this area, i.e. experiences of emerging research management/administration structures in other places (which would then later on support the conclusions drawn)
- findings from the pilot
- discussion of findings in context of the wider development of research management/administration globally
- conclusion/recommendation

Overall this is a very laudable and ambitious attempt at covering what seems a very large amount of work and data. The paper is also entering a field that overall still seems ‘under-researched’ with limited publications available. The paper would seem an important piece of work to contribute to the overall body of literature on evolving research support structures in HEIs. 

 The intent of this Open Letter might be to give an overview of the pilot study and some initial findings rather than embedding the work in a comprehensive sector and literature review. In this case a clearer focus on giving an introduction into the pilot and its aims and where possible sharing some initial findings might be a more realistic achievement in this paper. It would seem crucial to cross-reference and evidence any findings and conclusions.

I have read this submission. I believe that I have an appropriate level of expertise to confirm that it is of an acceptable scientific standard, however I have significant reservations, as outlined above.

# Peer review: Reviewer response for version 1

**DOI:** 10.21956/wellcomeopenres.16445.r34742

**Published:** 2019-02-14

**Authors:** Simon Kerridge

**Affiliations:** 1 University of Kent, Canterbury, UK

Overall the Open Letter is clear and compelling - India should invest in Research Management and Administration (RMA) infrastructure in order to better support researchers in their endeavours.  The specific recommendation for the creation of a national association for RMAs is also welcome.  However some of the assertions are not underpinned with evidence, at least not with evidence provided in the article.

 This is a real shame as the overall argument is (in my opinion) sound, but it is perhaps because of my experience as an RMA that I believe this, rather than with the evidence presented in the article - in some cases the Open Letter does not provide evidence for the assertions made.  However it does appear that much evidence will have been gathered in the IRMI work.  This could perhaps be more explicitly and directly drawn into the Open Letter – one method could be the incorporation of quotes from participants; but this could be problematic post hoc, and would perhaps change the tone of the article.  Another is to provide more detail on claims, for example “At present, Indian investigators spend a significant fraction of their time on administration…” there is no indication of what this fraction might be, or how the data to make the assertion was collected.

 In terms of unsupported assertions, another example is "Collaborative proposals involving Indian institutions lacking research offices often suffer delays, inadequate due diligence, undercosting of proposals on the Indian side, inadequate overheads and sluggish project management." - is this the case, where is the evidence?  It seems (to me) to be a reasonable assertion, and one I presume that came from the IRMI work – but the authors do not state this.  However, most of these assertions do reflect the findings of others, but again they are not referenced.

 India / IRMI specific assertions were similarly unsupported, for example “Support from a central office at the pre-award stages was found to be available at only a small minority of institutions.” How many of the 31 institutions looked at was this?  There is no underlying dataset to help answer this.

 One specific assertion that I do not quite follow is: “Individuals with backgrounds in areas such as science, medicine, dentistry and public health would likely play key roles in shaping RM structures for Indian institutions, in a manner that caters to specific institutional requirements and priorities.”  As shown in their 4 ^th^ reference (disclaimer, this was work I led), around the world, RMAs come from a wide variety of academic backgrounds.  It is not clear why this would be focussed around science and medicine in India.  However the IRMI work was based around institutions predominantly it seems in these subject areas and so perhaps, given the newness of the profession in India and the apparent propensity in countries where RMA is developing for RMAs initially to be researchers moving into administration, then perhaps this is to be expected.

 One specific weakness is in addressing opposing views.  This is only highlighted with the sentence “Lack of awareness also makes some researchers sceptical of the value of pre-award support, which was viewed as a hindrance or an administrative bottleneck.” which is then not countered or debated.  For example David Colquhoun makes some strong statements http://www.dcscience.net/DC-research-fortnight-020610.pdf; however the majority of the literature suggests that “good” research support can indeed unencumber the researcher from administrative burden, and even help improve the chances for research bids to be successful. See for example Pamela F. Miller (2017) ^1^, and Natasha G. Wiebe and Eleanor Maticka-Tyndale (2017) ^2^.

 Given the use of the worded “blended” in terms of professionalism, one might have expected a reference to the work of Celia Whitchurch.  In general there is a low level of citation.

 I would have liked to see “Research Management” and “RMA” included in the keywords.

 Overall the authors are to be commended in covering such a large amount of ground in such a short space, however this does perhaps mean that the reader has to take some assertions on face value.  Perhaps as an Open Letter, to provoke debate, this is not unreasonable but the arguments would be much stronger with the evidence base that the IRMI work surely produced – allowing the conclusions to be properly justified.

 In summary, the effectiveness of Research Management and Administration is in general an under researched area, and this Open Letter and its recommendations are welcomed, but is felt that some work is needed to show robust evidence for the conclusions made.

I have read this submission. I believe that I have an appropriate level of expertise to confirm that it is of an acceptable scientific standard, however I have significant reservations, as outlined above.

## References

[1] MillerPF: The Global Research Environment. *Chapter 3 in Research Management: Europe and Beyond*.2017;

[2] WiebeNGMaticka-TyndaleE: More and Better Grant Proposals? The Evaluation of a Grant-Writing Group at a Mid-Sized Canadian University. *Journal of Research Administration*.2107;67-92
